# Supplementary material for: Imaging of pH in vivo using hyperpolarized 13C-labelled zymonic acid
Source: Nat Commun. 2017 May 11;8:15126. doi: 10.1038/ncomms15126 (PMC5482723; doi:10.1038/ncomms15126)
Supplement: Supplementary Information — Supplementary figures and supplementary table. [file ncomms15126-s1.pdf]

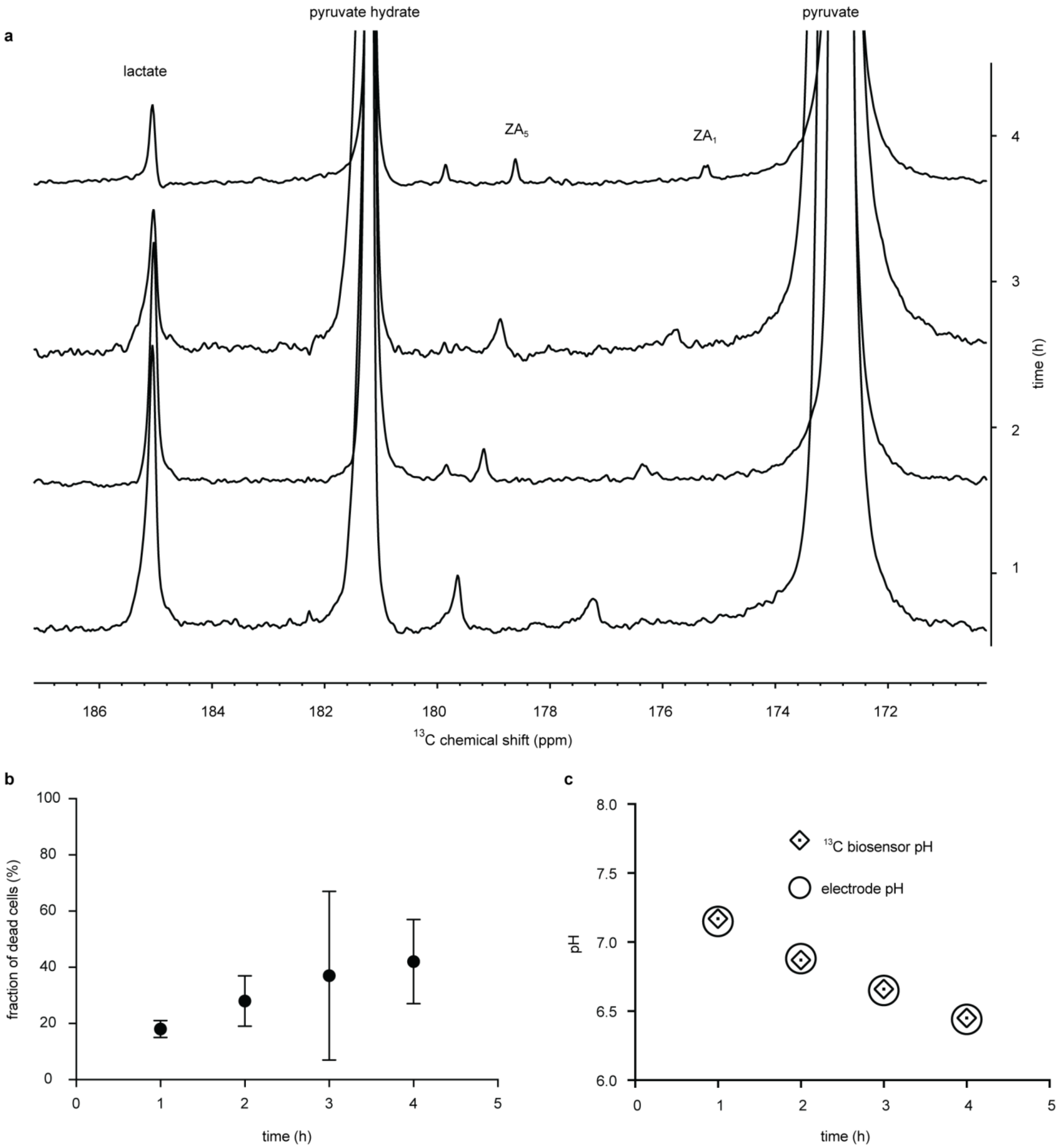

Supplementary Figure 1 | pH values determined by the hyperpolarized ^{13}C biosensor correlate with the extracellular pH in a tumor cell suspension at 14.1 T. Measurements in four samples (each at a different time t = 1, 2, 3, 4 h) of 2 mM hyperpolarized [1-^{13}C]pyruvate and 40×10^6 MCF-7 tumor cells being treated with Triton-X100 (at t = 0 h). (**a**) Pyruvate in aqueous solution is in equilibrium with pyruvate hydrate and exchanges its ^{13}C label with the lactate pool which in turn is enlarged within tumors by LDH activity. The presence of pyruvate led to trace amounts of detectable hyperpolarized ZA. (**b**) A treatment with Triton X-100 caused the fraction of dead cells to increase over time, determined by cell staining and fluorescence microscopy (see Methods). (**c**) The progressively increasing number of dead cells and anaerobic conditions over time led to a gradual acidification, with the ^{13}C biosensor pH correlating with the extracellular pH in the medium measured with a standard pH electrode after the NMR experiment.

a

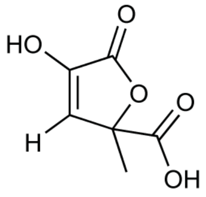

zymonic acid

Atom	δ (^{1}H) (ppm)	δ (^{13}C) (ppm)	J (^{1}H, ^{1}H) (Hz)	J (^{1}H, ^{13}C) (Hz)	J (^{13}C, ^{13}C) (Hz)
1		178.1		$^3J_{C1\text{-}H3}$=11.0	$^1J_{C1\text{-}C2}$=75, $^2J_{C1\text{-}C3}$=12
2		149.7			$^1J_{C2\text{-}C1}$=75, $^1J_{C2\text{-}C3}$=81
3	6.29 (s)	122.1		$^3J_{C3\text{-}H3}$=179.0, $^3J_{C3\text{-}H6}$=4.0	$^1J_{C3\text{-}C2}$=81, $^1J_{C3\text{-}C4}$=41, $^2J_{C3\text{-}C1}$=12
4		88.2		$^2J_{C4\text{-}H3}$=8.5, $^2J_{C4\text{-}H6}$=4.6	$^1J_{C4\text{-}C3}$=41, $^1J_{C4\text{-}C5}$=63, $^2J_{C4\text{-}C6}$=40
5		180.2		$^3J_{C5\text{-}H6}$=3.9	$^1J_{C5\text{-}C4}$=63
6	1.60 (s)	23.2		$^1J_{C6\text{-}H6}$=130.3	$^1J_{C6\text{-}C4}$=40

b

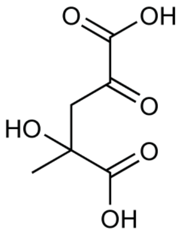

parapyruvic acid

Atom	δ (^{1}H) (ppm)	δ (^{13}C) (ppm)	J (^{1}H, ^{1}H) (Hz)	J (^{1}H, ^{13}C) (Hz)	J (^{13}C, ^{13}C) (Hz)
1		176.5			$^1J_{C1\text{-}C2}$=62, $^2J_{C1\text{-}C3}$=13
2		205.8			$^1J_{C2\text{-}C1}$=62, $^1J_{C2\text{-}C3}$=40
3	3.03 (d), 3.16 (d)	51.5	$^2J_{H3\text{-}C3}$=17.9		$^1J_{C3\text{-}C2}$=40, $^1J_{C3\text{-}C4}$=40, $^2J_{C3\text{-}C1}$=13
4		76.3			$^1J_{C4\text{-}C3}$=40, $^1J_{C4\text{-}C5}$=55, $^2J_{C4\text{-}C6}$=38
5		185			$^1J_{C5\text{-}C4}$=55
6	1.30 (s)	29			$^1J_{C6\text{-}C4}$=38

c

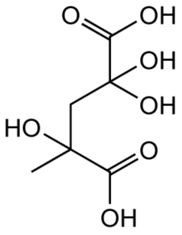

parapyruvic acid hydrate

Atom	δ (^{1}H) (ppm)	δ (^{13}C) (ppm)	J (^{1}H, ^{1}H) (Hz)	J (^{1}H, ^{13}C) (Hz)	J (^{13}C, ^{13}C) (Hz)
1		177.9			$^1J_{C1\text{-}C2}$=65, $^2J_{C1\text{-}C3}$=6
2		96.7			$^1J_{C2\text{-}C1}$=65, $^1J_{C2\text{-}C3}$=44
3	2.30 (d), 2.70 (d)	48.9	$^2J_{H3\text{-}C3}$=13.6		$^1J_{C3\text{-}C2}$=44, $^1J_{C3\text{-}C4}$=34, $^2J_{C3\text{-}C1}$=6
4		87			$^1J_{C4\text{-}C3}$=34, $^1J_{C4\text{-}C5}$=58, $^2J_{C4\text{-}C6}$=39
5		181.2			$^1J_{C5\text{-}C4}$=58
6	1.10 (s)	27			$^1J_{C6\text{-}C4}$=39

Supplementary Figure 2 | Chemical shifts and *J*-coupling constants extracted from NMR data acquired at 14.1 T. Fully ^{13}C-labeled ZA and its decay products dissolved in water reveal the chemical structures of all molecules observed during hyperpolarized experiments. Chemical shifts are unambiguously assigned to each molecule through their *J*-coupling networks. Shown are proton and carbon chemical shifts, as well as proton to carbon and carbon to carbon coupling constants of (**a**) zymonic acid, (**b**) parapyruvic acid and (**c**) parapyruvic acid hydrate in aqueous solution. (s), singlet. (d), doublet.

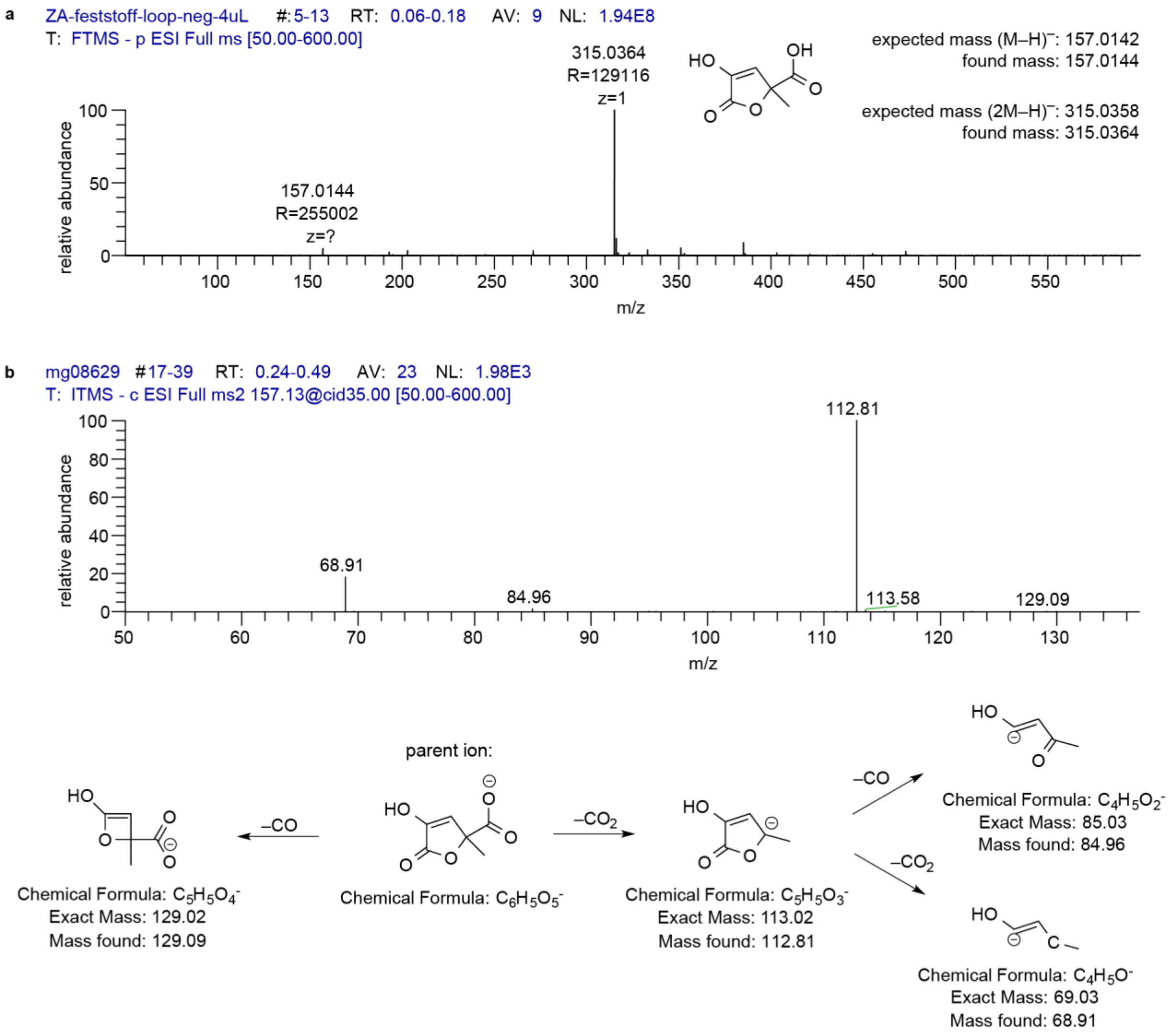

Supplementary Figure 3 | Confirmation of the structure of ZA using mass spectrometry. (**a**) The HR-MS-spectrum of the synthesized substance recorded with a Thermo Finnigan LTQ-FT confirms the total mass of the compound. (**b**) The MS/MS-spectrum of the synthesized substance recorded after CID-fragmentation on a Thermo Finnigan LCQ-Fleet and the putative assignment of the observed fragments is consistent with the structure. Within the accuracy of the ion trap (±0.3 m/z), all peaks can be explained by elimination of carbon monoxide and carbon dioxide.

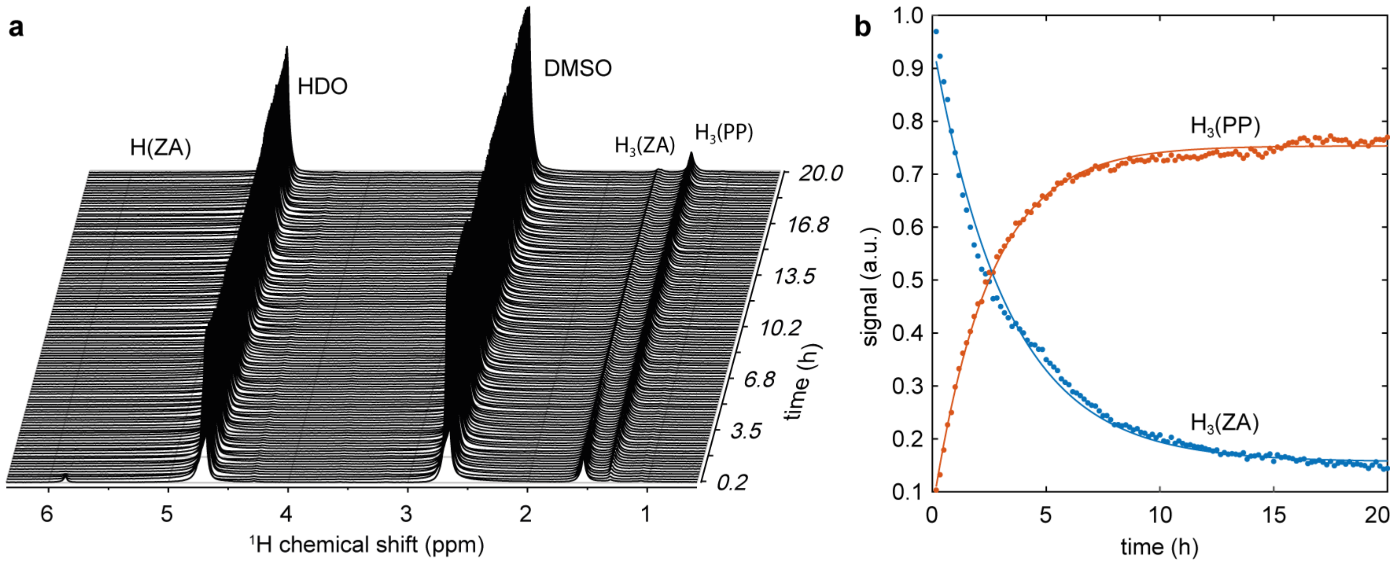

Supplementary Figure 4 | Stability of ZA in D_2O as a function of time. Slow chemical decay of ZA into parapyruvic acid (PP) in D_2O at pH = 7.54 ± 0.01. ^1H spectra were acquired at 1 T and 27 °C over 20 h, each spectrum averaged over 60 scans within 10 min. (**a**) The single proton H(ZA) attached to carbon ZA_3 can be seen at ~5.9 ppm, HDO at 4.7 ppm, DMSO at 2.7 ppm, the methyl group H_3(ZA) attached to carbon ZA_5 at 1.55 ppm and the methyl group H_3(PP) of parapyruvate hydrate at 1.35 ppm. The single proton attached to carbon ZA_3 is quickly exchanged for a deuteron by keto-enol-tautomerism and can thus only be observed in the first few spectra. (**b**) Peak amplitudes and exponentially fitted curves of the methyl groups of ZA and parapyruvic acid (PP) from **a** showing that ZA decays into PP with a half-life of $t_{1/2}$ = 2.27 ± 0.04 h.

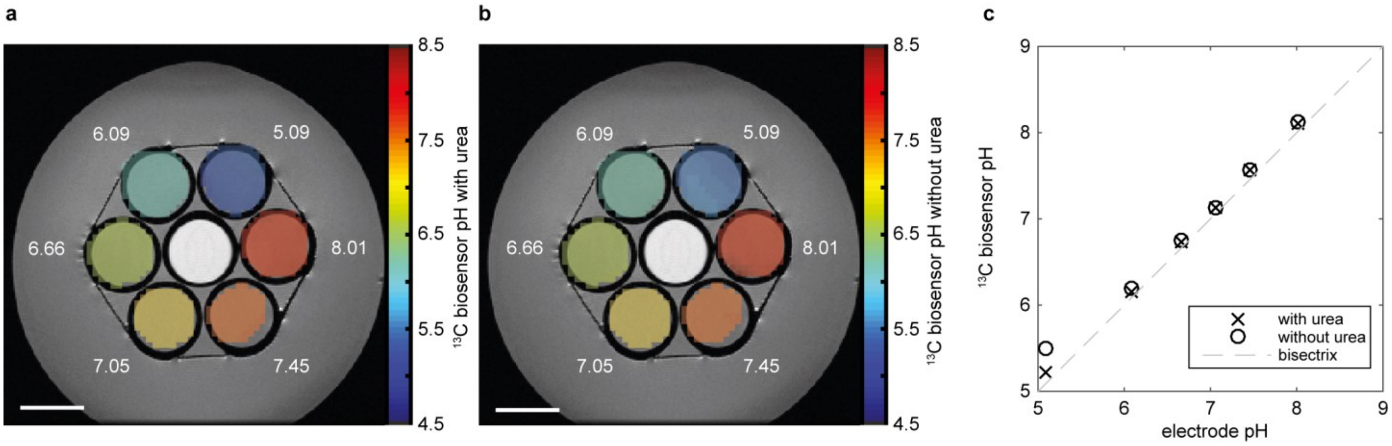

Supplementary Figure 5 | ^{13}C biosensor pH of the same buffer phantom measurement evaluated from ZA with and without considering the additional urea peak used as chemical shift reference at 7 T. The ^{13}C biosensor pH was back-calculated based on the chemical shift difference of both ^{13}C-labeled ZA positions (**a**) with respect to the pH insensitive ^{13}C urea and (**b**) based on the chemical shift difference between the two ^{13}C-labeled ZA positions only. (**c**) The pH values extracted from the two ^{13}C pH maps correlate well with the electrode pH (in white in **a** and **b**). At the limit of its sensitivity (at pH $\approx$ 5), the back-calculation of the ^{13}C biosensor pH is improved by taking the urea peak into account as pH insensitive chemical shift reference. Scale bars, 1 cm.

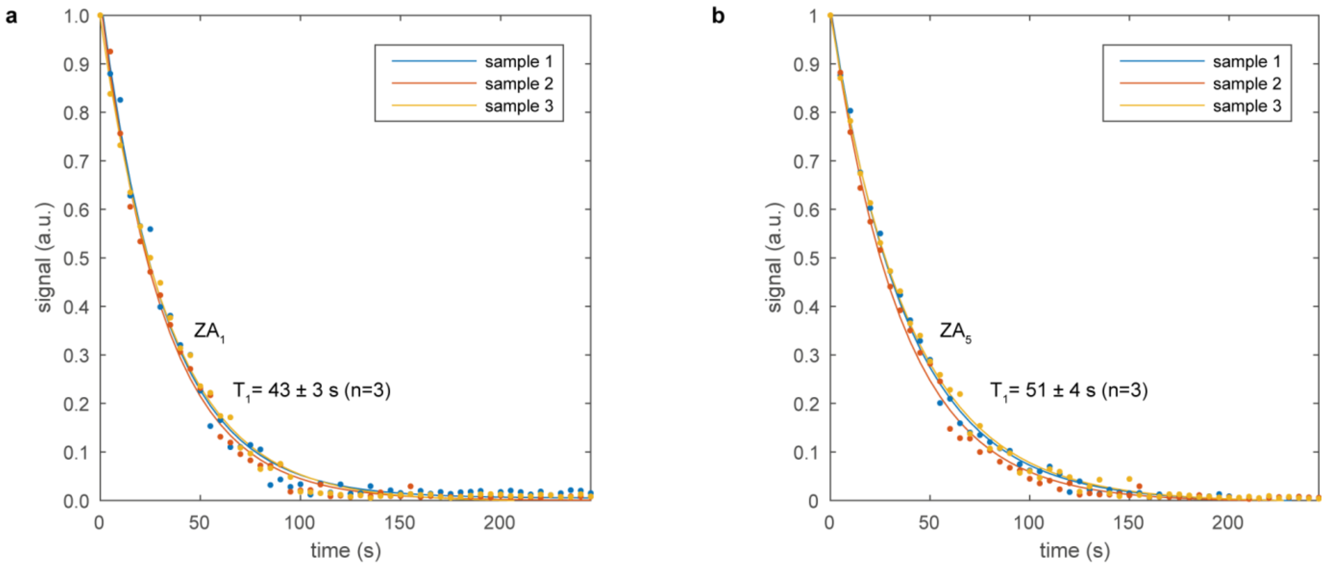

Supplementary Figure 6 | Longitudinal relaxation time T_1 of hyperpolarized natural abundance ZA *in vitro* at 3 T. A three-parameter monoexponential curve was fitted to each dataset and the mean and standard deviation was calculated from the resulting decay constants of 50 mM ZA in 80 mM Tris buffer in H_2O adjusted with 1M NaOH to an average pH of 6.53 ± 0.03 at 27 °C. The close proximity of the frequently and fast exchanging proton of the hydroxy group attached to carbon number two of ZA most likely causes the shorter T_1 of carbon number one ($^{13}ZA_{1,}$ **a**) compared to carbon number five ($^{13}ZA_5$, **b**) of ZA *in vitro*.

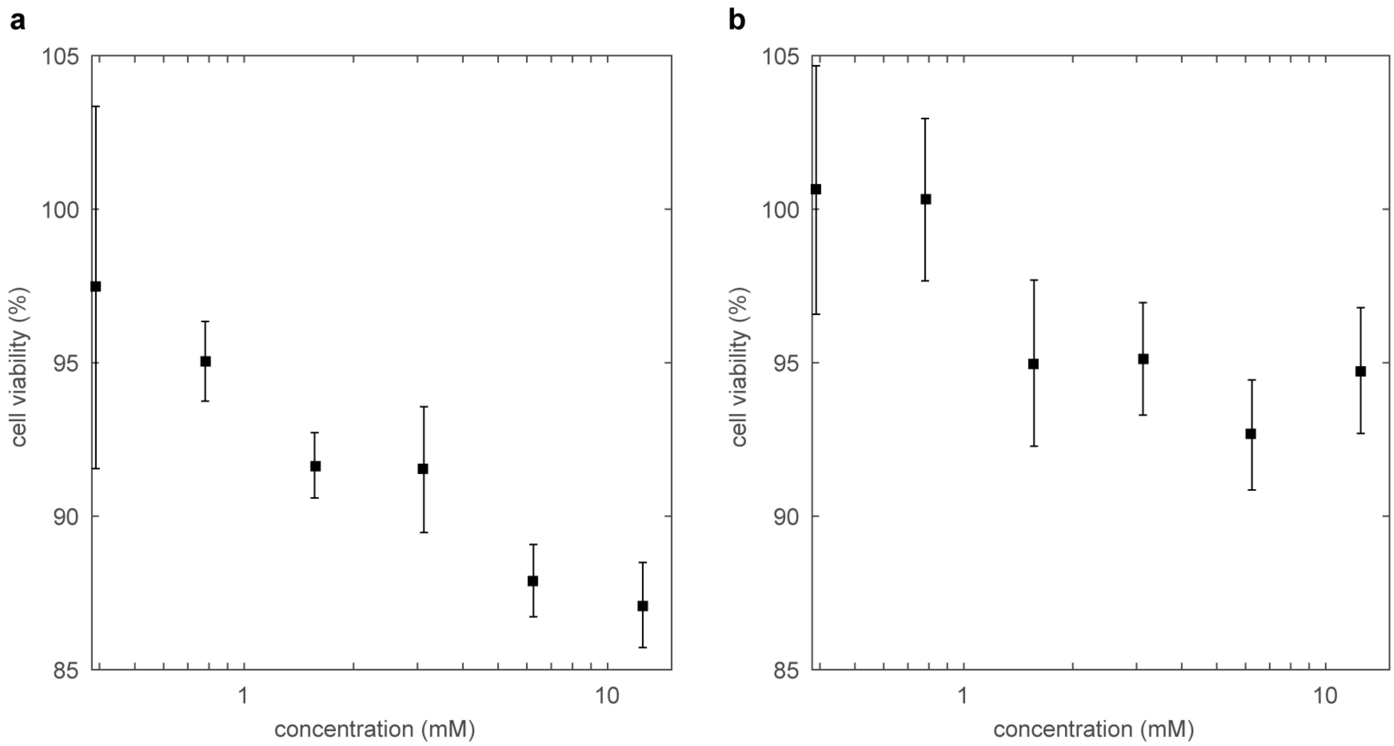

Supplementary Figure 7 | Cytotoxicity tests show that ZA is non-toxic within experimentally relevant concentration ranges. Typical concentrations of hyperpolarized substances injected into animals are on the order of 60–100 mM at a dose of approximately 5 mL kg^{-1}, resulting in an end concentration of the substance in the blood of 6–10 mM assuming a ratio of injected volume to blood volume in the order of 1:10. Therefore, 5000 HeLa cells each in 100 µL cell culture medium were incubated with ZA for 24 h at concentrations 0.4–12.5 mM (**a**) without and (**b**) with a Zn catalyst being used in the synthesis of ZA before being purified using reversed phase HPLC. No marked reduction in cell viability was observed (n = 3, mean ± s.d.).

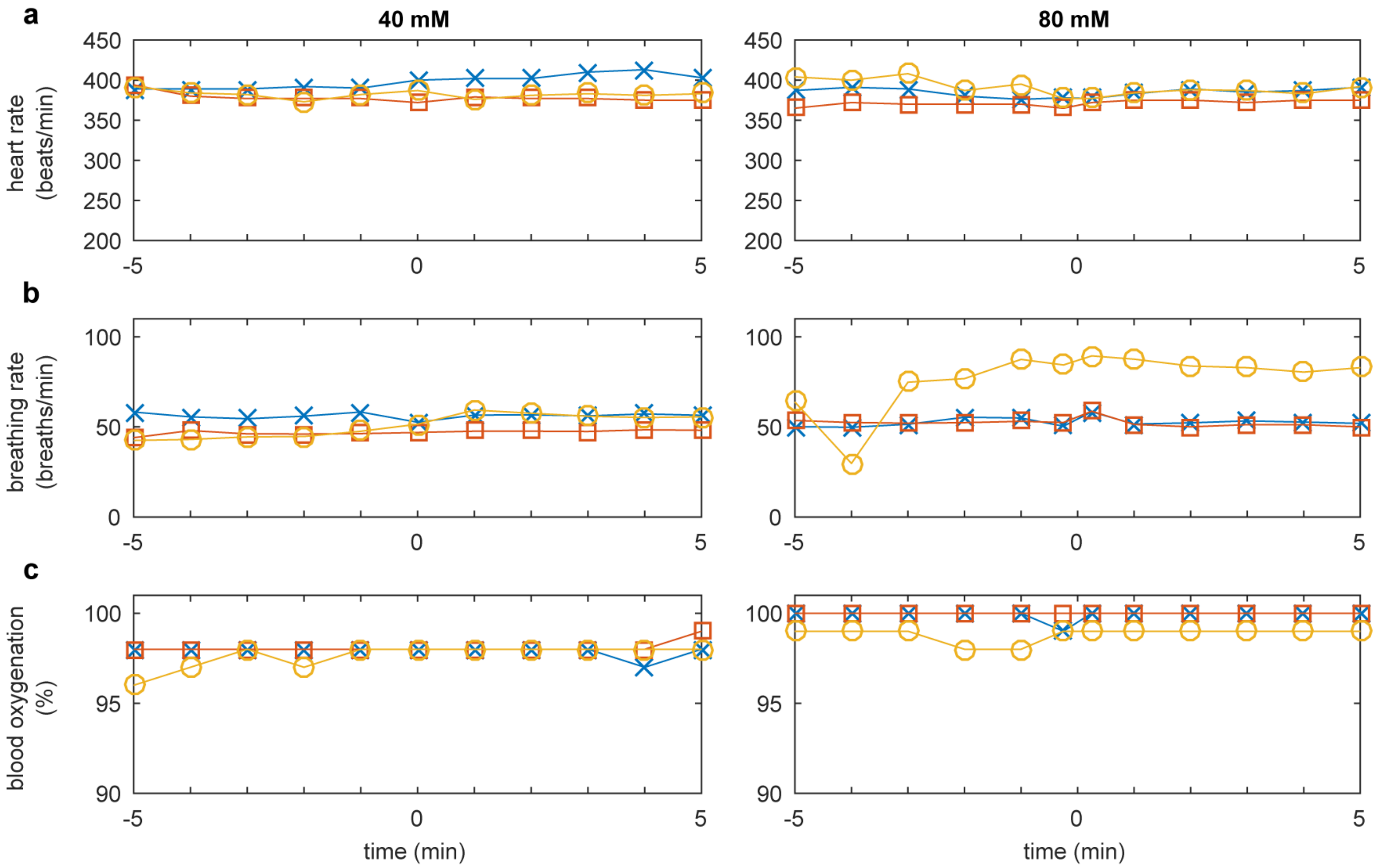

Supplementary Figure 8 | Dose escalation study testing for in vivo toxicity of ZA in three rats. ZA was dissolved in 80 mM Tris buffer solution, neutralized to normal blood pH ≈ 7.4 using NaOH, sterile filtered and injected into the tail vain at t = 0 min at a final injected concentration of 40 mM and 80 mM, at a dose of 5 mL kg^{-1} and a rate of 0.17 mL s^{-1}. For all three rats (Lewis, male, Charles River, average weight 319 ± 1 g), (**a**) heart rate, (**b**) breathing rate and (**c**) blood oxygenation were monitored for five minutes before and after injection. No abnormalities with respect to the injection of ZA were detected.

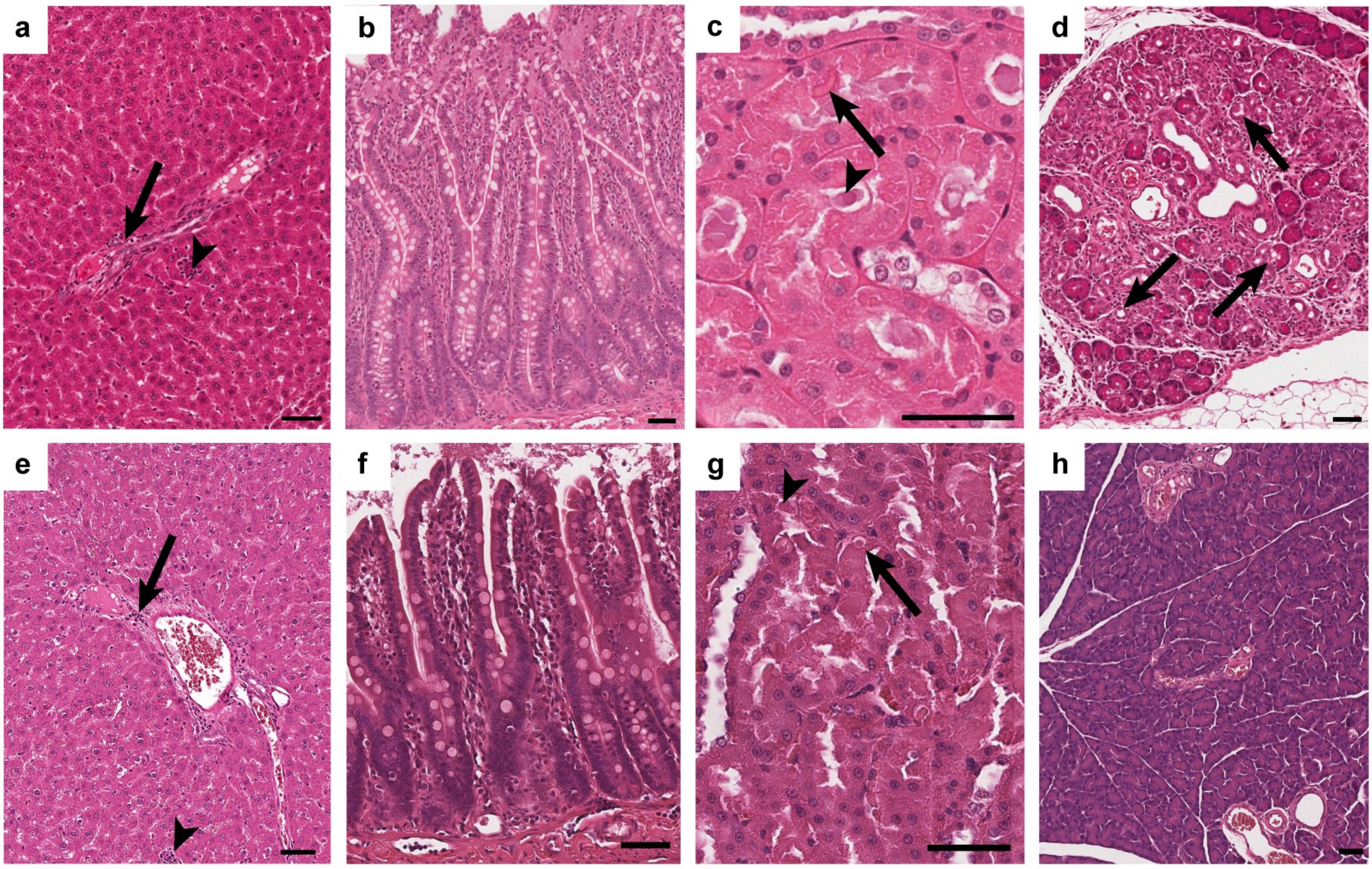

Supplementary Figure 9 | A toxicopathological study shows non-ZA-associated alterations both within exposed and unexposed animals. Representative images of background alterations observed histopathologically in (**a, e**) liver, (**b, f**) intestines, (**c, g**) kidney and (**d, h**) pancreas in animals after NaCl administration (upper row) and after fivefold overdosage of ZA (lower row). (**a, e**) In the liver, slight periportal infiltration predominantly with lymphocytes was observed in the periportal region (arrows) and intralobular (arrowheads). (**b, f**) Slight mixed infiltration and fibrosis of the villi occurred in all parts of the intestines. (**c, g**) In the kidney, intraepithelial (arrows) and intraluminal (arrowheads) hyaline droplets within the proximal tubuli were observed only in male rats regardless of the injected compound. (**d**) One of the control animals showed a focal acinar-to-ductular metaplasia within the pancreas. The arrows indicate metaplastic ductular formations. (**h**) Normal pancreatic tissue in an animal after fivefold overdosage of ZA. H&E staining. Scale bars, 50 µm.

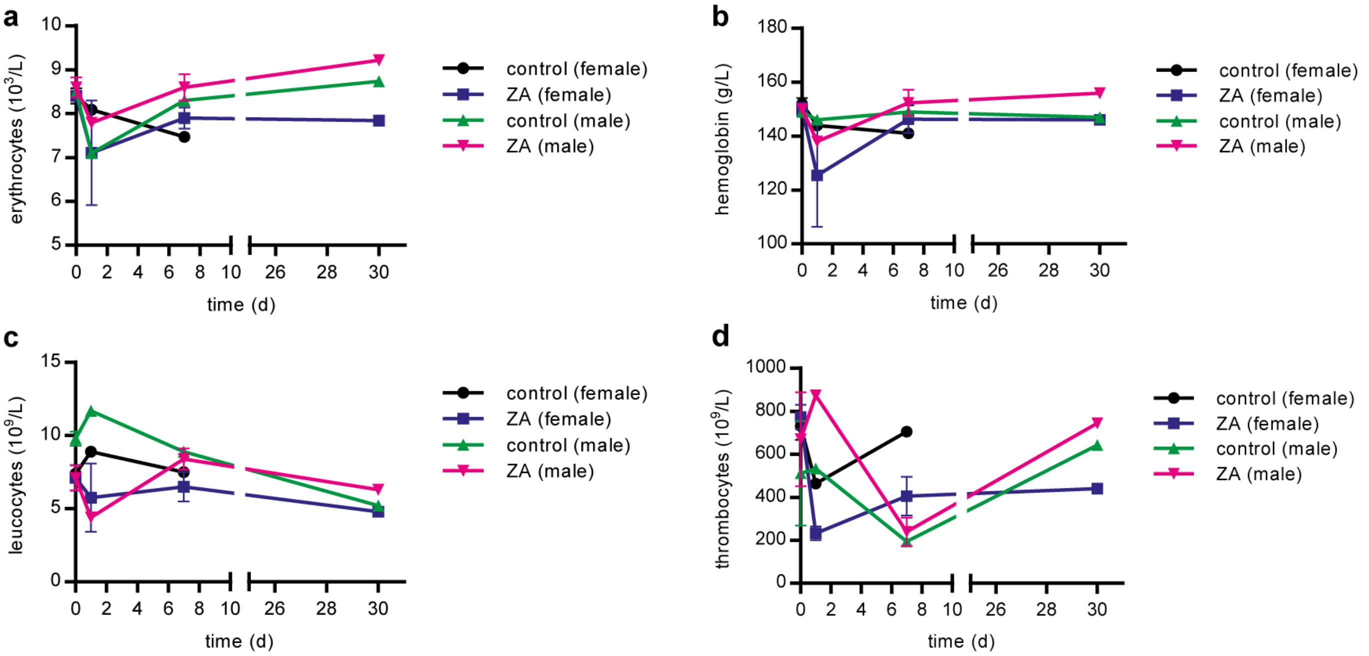

Supplementary Figure 10 | Non-toxicity of ZA was substantiated by blood collection for hematology and clinical chemistry before and 24 hours (acute), 7, 21 and 30 days (subacute) after injection of ZA. Here, four representative mean values of the blood analysis are shown: (**a**) erythrocytes, (**b**) hemoglobin, (**c**) leucocytes, and (**d**) thrombocytes. All levels are close to the reference levels (erythrocytes: 5.5–9.3 × 10^3 L^{-1}, hemoglobin: 106–156 g L^{-1}, leucocytes: 3.3–8.7 × 10^9 L^{-1}, thrombocytes: 500–1300 × 10^9 L^{-1}) provided by the supplier (Charles River) and no significant difference between ZA and NaCl injected animals can be detected. 14 animals (7 female / 7 male) were used, 10 of them received a tail vein injection of 5 mL kg^{-1} with a concentration of 250 mM ZA (5 times the dosage used for the imaging experiments), 4 animals served as controls with a tail vein injection of saline (0.09 % w/v of NaCl) (see also Methods).

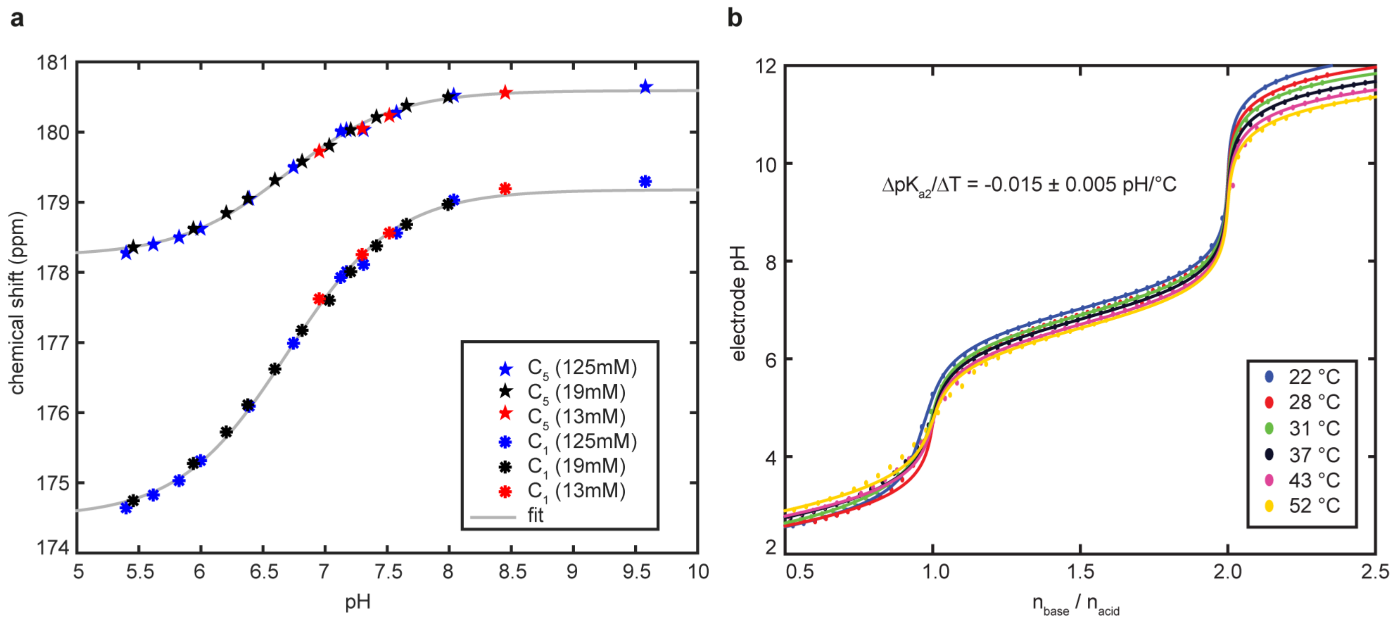

Supplementary Figure 11 | Concentration and temperature dependence of the pH detection using ZA. (**a**) Chemical shifts as a function of pH are independent of the concentration of ZA within experimentally relevant concentration ranges. ^{13}C chemical shifts of ZA_5 and ZA_1 at starting concentrations of 125 mM, 19 mM and 13 mM ZA at 14.1 T and 37 °C in 1 M phosphate buffer, titrated in random order to different pH values using 1 M HCl and 1 M NaOH. The fast proton exchange mechanism leading to changes in chemical shift as a function of pH is independent of the concentration of ZA itself. (**b**) ZA shows a weak temperature dependence of the relevant acid dissociation constant pK_{a2}. A 25 mM solution of ZA in 5 mL 1 M KCl in H_2O was titrated with 1 M KOH at different temperatures. Each full titration was performed within 22 ± 1 min and a theoretical titration curve for a diprotic acid was fitted to each titration experiment. Evaluating the relevant extracted acid dissociation constant pK_{a2} as a function of temperature results in a weak temperature dependence of $\Delta pK_{a2} / \Delta T = -0.015 \pm 0.005$ pH / °C, making ZA a temperature independent pH reporter within biologically relevant temperatures.

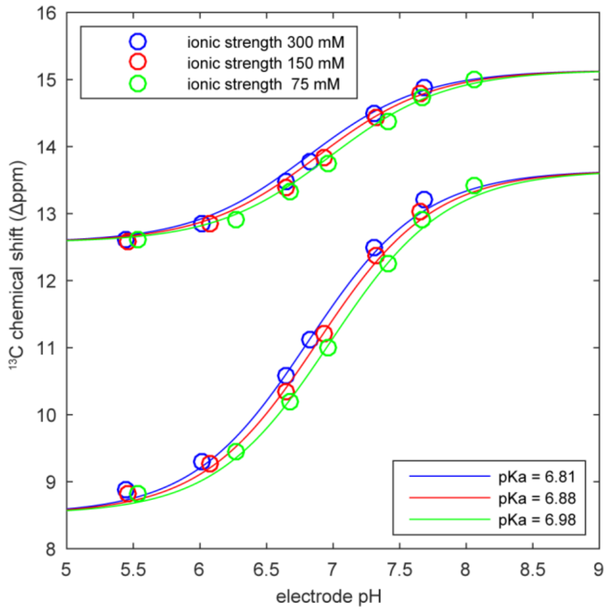

Supplementary Figure 12 | Thermal ^{13}C chemical shift as a function of electrode pH at three different ionic strengths at 5.9 T. 20 mM [1,5-$^{13}C_2$]ZA and 20 mM ^{13}C urea in KCl solution of varying ionic strength containing 10 % D_2O were titrated with 2.5 M KOH. The total given ionic strength is the sum of the ionic strength of KCl and single charged zymonic acid. The measurements were performed at 37 °C and 5.9 T. From a linear fit to the fitted acid dissociation constant pKa as a function of ionic strength I one finds $\Delta pKa / \Delta I = -0.7 \times 10^{-3}$ pH / mM, so that the pH uncertainty in the physiological range with an ionic strength of 135–165 mM results in ≈ 0.02 pH units.

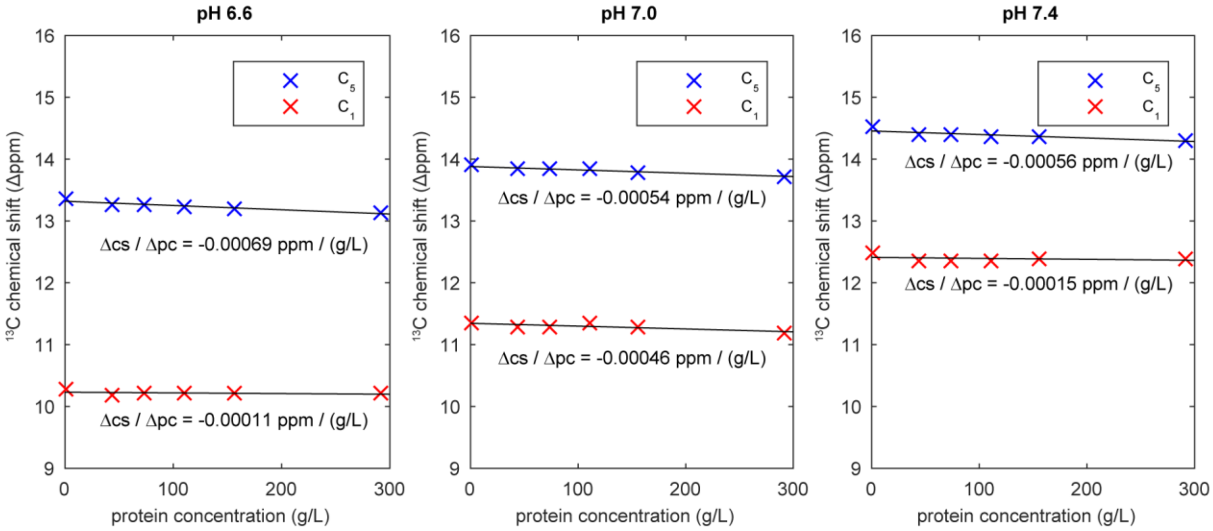

Supplementary Figure 13 | Thermal ^{13}C chemical shift of [1,5-$^{13}C_2$]ZA at three different pH values as a function of protein concentration at 5.9 T. 20 mM [1,5-$^{13}C_2$]ZA and 20 mM ^{13}C urea in 130 mM KCl and 10% D_2O were set to the pH values 6.6, 7.0 and 7.4 at 37 °C. Increasing amounts of bovine serum albumin (BSA) were added and the pH was readjusted before each individual measurement. From the linear fits we detected a maximum change in chemical shift cs as a function of protein concentration pc of $\Delta cs / \Delta pc = -0.7 \times 10^{-3}$ Δppm / (g L^{-1}), so that the pH uncertainty in the physiological range with a protein concentration of 60–80 g L^{-1} results in ≈ 0.01 pH units.

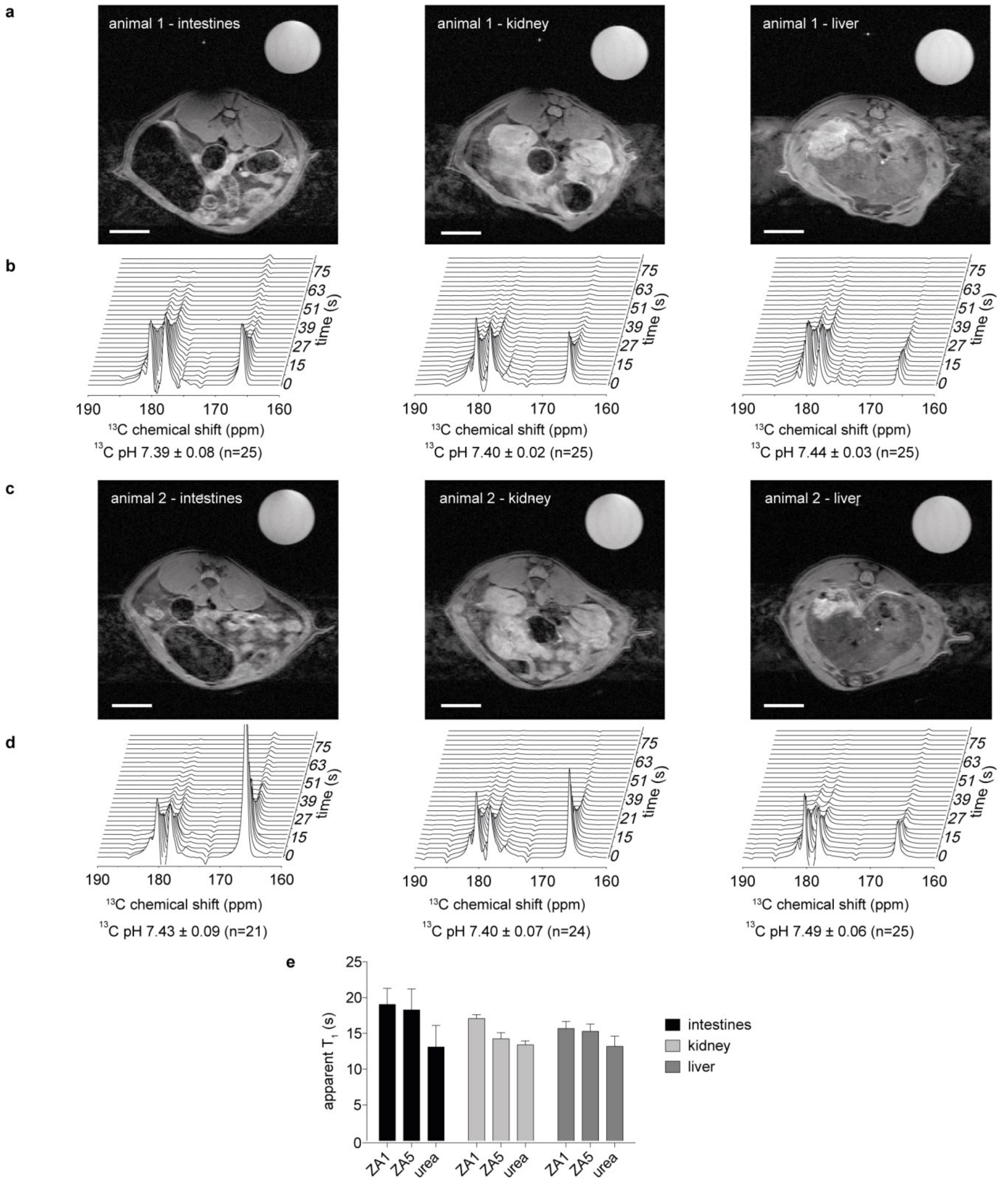

Supplementary Figure 14 | Apparent longitudinal relaxation time T_1 of hyperpolarized ZA *in vivo* at 7 T. (**a**, **c**) Proton anatomical images of the three axial slices through intestines, kidney and liver where pH and T_1 measurements were performed within two animals. (**b**, **d**) Time resolved slice selective spectra of hyperpolarized ZA and ^{13}C urea report consistent pH values within the physiologically expected pH range after tail vain injection. (**e**) Monoexponential decay curves fitted to the peak maxima of ZA_1, ZA_5 and urea result in slightly longer apparent T_1 times for ZA compared to urea and no significant differences in apparent T_1 between the three slices. For each *in vivo* experiment, spectra from 64 time steps were recorded using a flip angle of 10 ° and a TR of 3 s. The pH values were calculated from the difference of the chemical shifts of ZA_1, ZA_5 and urea. The ^{13}C pH values are given as mean ± standard deviation of the *n* spectra that could be used for the back-calculation of the pH due to sufficient SNR. Scale bars, 1 cm.

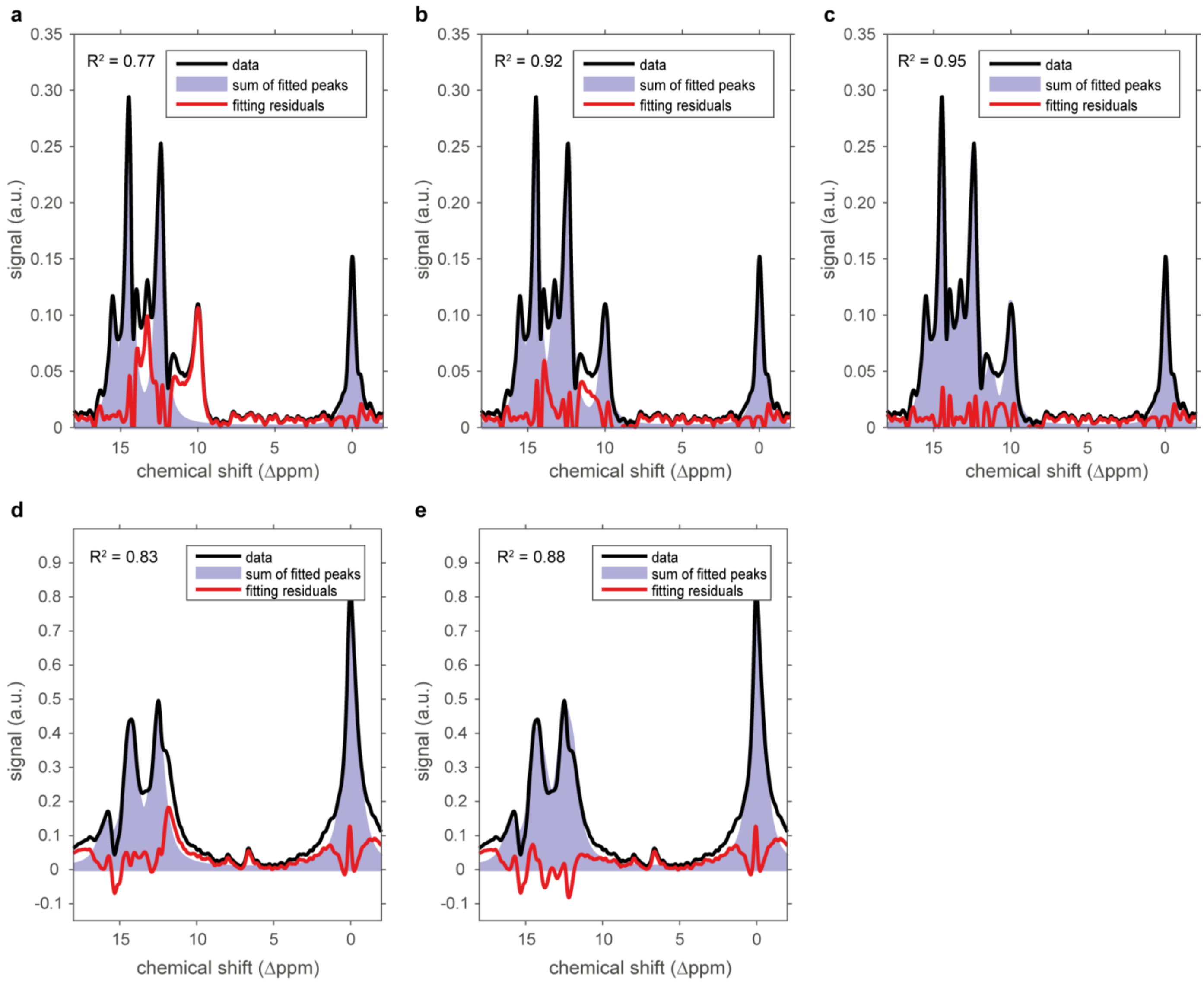

Supplementary Figure 15 | Representative fits and fitting residuals for multiple tissue compartments in the kidneys and in the tumor. (**a-c**) In the kidneys, increasing the number of fitted zymonic acid peak pairs from one (**a**, R^2 = 0.77) to two (**b**, R^2 = 0.92) to three (**c**, R^2 = 0.95) results in a reduction of the fitting residuals (red line) and an improved coefficient of determination R^2. (**d-e**) Analogously, increasing the number of fitted zymonic acid peaks pairs from one (**e**, R^2 = 0.83) to two (**f**, R^2 = 0.88) results in a reduction of the fitting residuals (red line) and an improved coefficient of determination R^2 in the tumor. Urea (0 ppm) and parapyruvate hydrate (15.7 ppm) are fitted in all spectra.

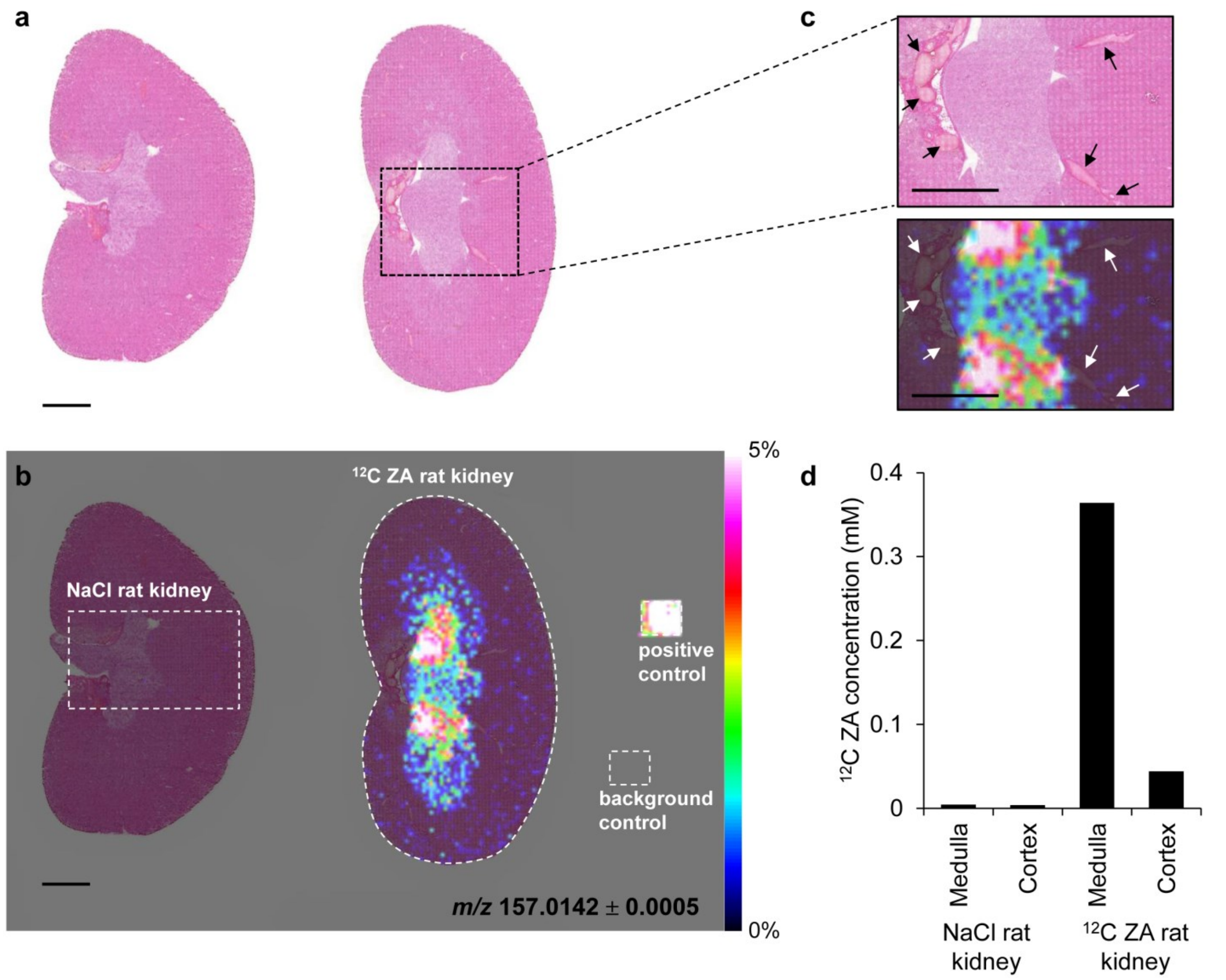

Supplementary Figure 16 | Distribution of ^{12}C ZA in rat kidney and extravasation into kidney tissue was confirmed by matrix-assisted laser desorption/ionization mass spectrometry imaging (MALDI-MSI). (**a**) H&E stains of analyzed tissue sections. (**b**) Distribution of ^{12}C ZA (*m/z* 157.0142) in kidney section 30 seconds after injection of 5 mL kg^{-1} 250 mM ^{12}C-ZA. The control rat was administered with isotonic saline. A positive control, 1 mM ^{12}C-ZA droplet on slide, and a non-tissue measurement region acting as a background control were included in the measurement. All samples were coated with 9-aminoacridine and analyzed in negative mode on a FT-ICR MS. Data was acquired at a spatial resolution of 150 µm and normalized by root mean square. Dashed lines mark the measurement regions. (**c**) Higher magnification image showing arterial and venous blood vessels (black and white arrows) and high abundant signals of ZA in the medulla of the kidney. (**d**) Mean ^{12}C ZA concentrations in renal cortex and medulla. MALDI-MSI represents the distribution of ZA within the kidney fixed 2-3 minutes after injection whereas hyperpolarized MRI shows the distribution of ZA within the kidney 10 s after injection. Whereas in the hyperpolarized MR image, shortly after injection, the cortex exhibits the largest contribution to the overall signal, in MALDI-MSI, much longer after injection, more ZA is already involved in the renal filtering process and thus the area containing the medulla and calyx show the largest signal contribution. Scale bars, 2 mm.

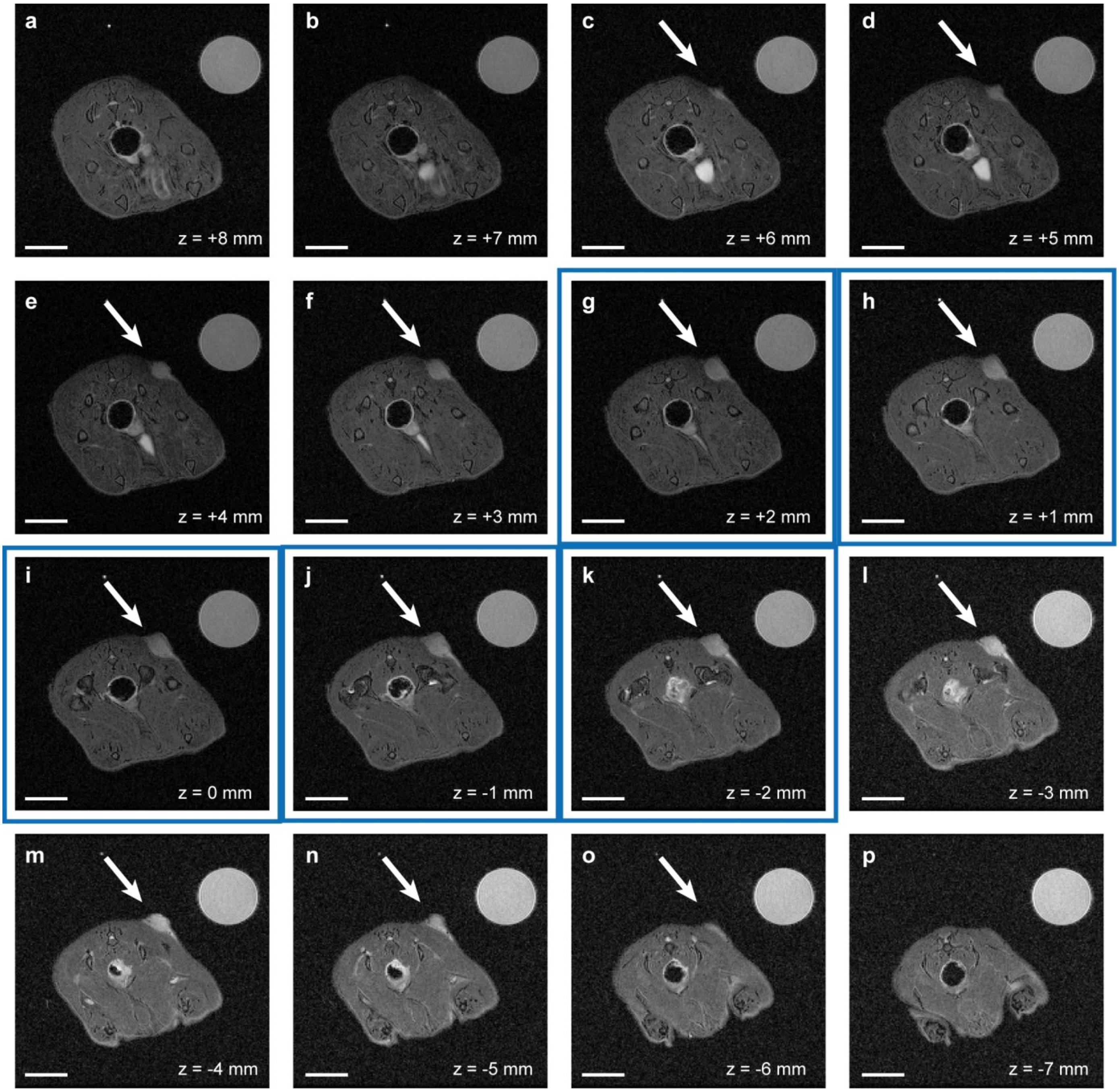

Supplementary Figure 17 | Axial slices from the animal shown in Fig. 5 bearing a Mat B III tumor (arrow). **(a-p)** Proton images with a field of view of 6 cm were acquired every 1 mm using a fast spin echo sequence (see Methods). **(g-k)** The five proton images contained within the 5 mm thick hyperpolarized ^{13}C image are marked with a blue box. Image **(i)** represents the central tumor slice and coincides with the center of the 5 mm thick hyperpolarized ^{13}C image. Scale bars, 1 cm.

	NaCl 24h		ZA 24h		NaCl 4 weeks		ZA 4 weeks	
	**female**	**male**	**female**	**male**	**female**	**male**	**female**	**male**
**Liver**								
inflammation, focal, slight	1/1	0/1	1/2	1/2	1/1	3/3	1/1	3/3
fatty change, macrovesicular, zone 3, slight	0/1	0/1	0/2	1/2	0/1	0/3	0/1	0/3
**GI**								
inflammation, mixed, diffuse, with fibroplasia, slight	1/1	1/1	2/2	2/2	1/1	3/3	1/1	3/3
**Kidney**								
accumulation, hyaline droplets, proximal tubules, slight/moderate	0/1	1/1	0/2	2/2	0/1	1/1	0/3	3/3
**pancreas**								
metaplasia, ductular, focal, slight	0/1	0/1	0/2	0/2	1/1	0/1	0/3	0/3

Supplementary Table 1 | Toxicopathological study. Distribution of histopathological alterations after a single administration of ZA at 5 times the dosage used for the imaging experiments, compared to NaCl administration after 24 h and 4 weeks, respectively. Overall, no significant histopathological difference between the ZA and NaCl injected animals could be detected.
